# Expanding the health belief model on dementia knowledge, fear, and preventive behaviors among older adults in Korea: a cross-sectional descriptive study

**DOI:** 10.7586/jkbns.25.008

**Published:** 2025-02-24

**Authors:** Jeong Eui Yun, Suyoung Choi

**Affiliations:** 1 Jeju Eastern Center for Dementia, Jeju, Korea; 2 College of Nursing, Health and Nursing Research Institute, Jeju National University, Jeju, Korea

**Keywords:** Aged, Dementia, Health belief model, Knowledge

## Abstract

**Purpose:**

This study aimed to expand the health belief model by incorporating dementia knowledge and fear and to assess the effects of these variables on dementia prevention behaviors among older adults.

**Methods:**

In total, 199 elderly individuals from 10 senior centers in Korea completed a structured questionnaire assessing characteristics, dementia knowledge, fear, health beliefs, and prevention behaviors. Measures included the Alzheimer’s Disease Knowledge Scale, the Korean version of the Fear of Alzheimer’s Disease Scale, and the Korean version of the Motivation to Change Lifestyle and Health Behavior for Dementia. Hierarchical polynomial regression was conducted to examine the impact of integrating dementia knowledge and fear into the health belief model.

**Results:**

Hierarchical polynomial regression across four models revealed significant effects of various factors on dementia prevention behaviors, explaining 12%–36% of the variance. Perceived barriers significantly decreased dementia prevention behaviors, while higher levels of self-efficacy and cues to action had a positive influence. Additionally, a nonlinear relationship was identified between dementia knowledge and prevention behaviors, with the positive impacts of increased knowledge potentially diminishing beyond a certain point.

**Conclusion:**

Our findings underscore the necessity of modifying the health belief model to integrate dementia knowledge and fear, which play critical roles in shaping preventive behaviors among older adults. Future investigations should examine the optimal level of dementia knowledge to promote these behaviors and elucidate the intricate relationship between knowledge and actions.

## INTRODUCTION

Globally, approximately 50 million people suffer from dementia, with a new case occurring every three seconds. By 2050, the number of people with dementia is expected to triple [1]. In Korea, dementia prevalence among individuals aged ≥ 65 years was 10.4% in 2020, affecting approximately 980,000 individuals. This number is projected to rise to 16.6%, or approximately 3.14 million individuals, by 2050, further increasing the societal and economic burden [2]. Dementia not only affects patients but also imposes significant caregiving and financial burdens on families and society. The global cost of dementia-related healthcare was estimated at United States dollar 1 trillion in 2010 and is expected to increase by 85% by 2030. In Korea, the annual cost of dementia management was estimated at 20.8 trillion Korean won (KRW) in 2022, representing 1% of the national gross domestic product, with projections reaching 138.1 trillion KRW by 2050 [3].

Dementia, which is characterized by acquired cognitive impairments that hinder daily and social functioning, has currently no available cure. Therefore, preventive management to delay its onset is essential. The World Health Organization has emphasized the importance of lifestyle changes, such as healthy eating, regular exercise, smoking cessation, and alcohol moderation, as well as the management of cardiovascular risk factors, such as obesity, hypertension, and diabetes. Furthermore, mental health care and early detection of dementia are critical [1]. In line with this, the Ministry of Health and Welfare of South Korea had established its 4th Comprehensive Dementia Management Plan (2021–2025), which focuses on expanding dementia screening to all ages and managing suspected cases through public health initiatives [4].

The health belief model (HBM), which had been developed to explain individuals’ preventive health behaviors, posits that perceived threats of illness (perceived susceptibility and perceived severity), expected outcomes of health behaviors (perceived benefits and barriers), and specific cues to action motivates individuals to engage in health-promoting actions [5]. Subsequently, the model was expanded to include the concept of health motivation to better explain patients’ role-related behaviors [5]. Additionally, as health behaviors began to focus on long-term lifestyle changes, the concept of self-efficacy was incorporated into the model to enhance the explanatory power of the model for preventive behaviors [6,7].

However, the HBM has been criticized for failing to include significant emotional factors, such as fear, which can play a crucial role in motivating behavioral changes and adopting preventive health behaviors [8]. Although previous studies have explored the relationship between dementia-related health beliefs and preventive behaviors, integration of emotional factors, such as fear, remains limited [9]. Additionally, only a handful of studies have incorporated knowledge of dementia into HBM [10]. Dementia knowledge refers to the extent of an individual’s understanding of various aspects related to dementia, including its causes, symptoms, treatment, and prevention [11]. Individuals with higher levels of dementia knowledge enables can accurately comprehend their condition, fostering appropriate perceptions and attitudes that positively influence dementia-preventive behaviors [11]. Conversely, a lack of dementia knowledge may promote unhealthy preventive actions and lifestyle choices, delaying the diagnosis of dementia and negatively affecting treatment outcomes [11]. Addressing this gap could provide new insights into promoting preventive behaviors among older adults, particularly in a super-aged society. This study aimed to expand the HBM by incorporating dementia knowledge and fear and evaluate their impact on dementia prevention behaviors among community-dwelling older adults.

## METHODS

### 1. Study design

This descriptive cross-sectional study investigated factors influencing dementia prevention behaviors among community-dwelling older adults with the specific aim of expanding the HBM by integrating variables, such as dementia-related knowledge and fear.

### 2. Participants

Our study targeted adults aged 65 years and above residing in Jeju, Korea, recruited from 10 senior centers through convenience sampling. Inclusion criteria were: (1) age ≥ 65 years, (2) Korean cognitive impairment screening test (CIST) results indicating normal cognitive function as assessed by the Jeju Center for Dementia, and (3) the ability to comprehend and respond to survey questions. The cut-off score for normal cognitive functions in the CIST, used by the Center for Dementia, is applied according to criteria established by the Ministry of Health and Welfare, varying by age and educational level. The sample size was determined using G-Power 3.1.9.2 [12] for multiple regression analysis with a medium effect size (0.15), α = .05, power (1 − β) = .80, with 21 predictors (12 sociodemographic characteristics, dementia knowledge, dementia fear, and seven dementia health beliefs). A minimum of 160 participants was necessary, according to the analysis. Accounting for an anticipated dropout rate of 20% to 30%, we aimed to recruit between 200 and 229 participants. Ultimately, we enrolled 204 participants, of whom 199 (97.5%) completed the survey.

### 3. Instruments

This study used a structured questionnaire that consisted of 115 items on sociodemographic characteristics (12 items), dementia knowledge (30 items), dementia fear (27 items), dementia health beliefs (27 items), and dementia prevention behaviors (16 items).

#### 1) Participants’ characteristics

Participant characteristics were categorized into general and dementia-related. General characteristics included age, sex, living arrangements, religion, education level, monthly income, number of chronic diseases, and perceived health status. Dementia-related characteristics consisted of a family history of dementia, experience in caregiving for family members with dementia, experience in dementia prevention education, and plans for dementia screening.

#### 2) Dementia knowledge

This study utilized the Korean version of the Alzheimer’s disease knowledge scale (ADKS) [13], adapted to and validated for used among the Korean population [14]. This scale measures dementia knowledge across risk factors, diagnosis, symptoms, progression, treatment, and care, with a total score ranging from 0 to 30. Given that the ADKS is designed to assess general knowledge rather than underlying psychological constructs or dimensions, internal consistency measures such as Cronbach’s alpha are less relevant. Instead, test-retest reliability is crucial for ensuring stability across different groups and time points [15]. Therefore, we assessed reliability using the intraclass correlation coefficient (ICC) with a two-week interval between tests. The test–retest reliability of the original tool was .81 [13], whereas the ICC in this study was .83 (95% confidence interval: .75~.89).

#### 3) Dementia fear

Dementia fear was measured using the Korean version of the fear of Alzheimer’s disease scale (K-FADS), adapted by Moon et al. [16] from the original tool (the FADS) [17] . The K-FADS assesses the general fear of dementia, fear related to physical symptoms, and catastrophic attitudes toward Alzheimer’s disease using a 5-point Likert scale. Regarding reliability, the original FADS tool had a Cronbach’s α value of .94 [17], whereas the K-FADS demonstrated a Cronbach’s α value of .98 [16]. In this study, Cronbach’s α for the K-FADS was .95.

#### 4) Dementia health beliefs

Dementia health beliefs were measured using the Korean version of the Motivation to Change Lifestyle and Health Behavior for Dementia Reduction scale [18], adapted from the original [19]. This tool assesses perceived susceptibility, severity, benefits, barriers, health motivation, cues to action, and self-efficacy using a 5-point Likert scale. The original scale's Cronbach’s α values ranged from .61 to .86 [19], while this study reported Cronbach’s α values of .68 to .98.

#### 5) Dementia prevention behaviors

Dementia prevention behaviors were measured using a modified version of the assessment tool by Jung and Gu [9], which was originally developed by Lim et al. [20]. Lim’s tool is based on three categories: recommended behaviors (moderate-intensity exercise for at least 30 min, balanced consumption of nuts and vegetables, three meals a day, sufficient sleep, reading, and writing), avoidance behaviors (alcohol consumption, smoking, brain injury, obesity, and chronic disease), and behaviors to practice (stress relief, depression prevention, social interaction, early dementia screening, and regular health check-ups) [20]. Jung and Gu [9] added one item on hand exercises, modifying the tool based on the Ministry of Health and Welfare’s 10 dementia prevention recommendations. This tool uses a 5-point Likert scale, with higher scores indicating more frequent performance of dementia prevention behaviors. The reliability of the original tool was Cronbach’s α = .77 [20], Jung’s study reported a Cronbach’s α value of .79 [9], and this study confirmed the Cronbach’s α to be .75.

### 4. Data collection

Data were collected between August and October 2022. Participants were recruited from 10 senior centers in Jeju, where the Jeju Center for Dementia had conducted the CIST within the past year. Researchers visited the senior centers during briefing sessions organized by the Jeju Center for Dementia, which were held to promote their dementia prevention program for cognitively normal elderly individuals identified through prior screenings. After obtaining a permit from the Jeju Center for Dementia and institutional approval, the researchers visited the senior centers to explain the study’s purpose and procedures. Written informed consent was obtained from all participants. For participants with visual impairments or literacy issues, researchers conducted face-to-face interviews to administer the survey.

### 5. Data analysis

Data were analyzed using SPSS version 23.0 (IBM Corp., Armonk, NY, USA). Among the 204 participants identified, 199 were analyzed after excluding five insincere respondents. The frequencies, percentages, means, and standard deviations were calculated for participant characteristics and survey scores. Internal consistency was assessed using Cronbach’s α, and test–retest reliability was measured with the ICC. Independent t-tests and one-way analysis of variance examined differences in dementia prevention behaviors according to participant characteristics, with post-hoc analyses using Scheffé’s test. Pearson correlation coefficients explored relationships between dementia knowledge, fear, health beliefs, and prevention behaviors. To identify predictors of dementia prevention behaviors and evaluate the nonlinear effects of dementia knowledge, we employed a hierarchical polynomial regression model. Prior to this analysis, mean centering was applied to dementia knowledge and its quadratic term to effectively reduce multicollinearity, ensuring the reliability of our regression results.

### 6. Ethical considerations

This study was approved by the Institutional Review Board (IRB) of Jeju National University (IRB No. 2022-052) and complied with the Declaration of Helsinki. After obtaining permission from the Jeju Center for Dementia, we collected data only on participation eligibility based on their assessments rather than specific CIST scores. This approach was explicitly stated during the IRB review process. Each participant was provided with a document elucidating the background and objectives of the research, contents of the survey, advantages of participation, assurance of confidentiality, protocols for data storage and destruction, and the consent process for study participation. Participants were informed of their right to withdraw from the study at any time and were given detailed information regarding the researchers. After obtaining written consent, the participants were presented with a modest gift as an expression of appreciation.

## RESULTS

### 1. Influence of participants’ characteristics on dementia prevention behaviors

The mean age of the participants was 76.36 ± 7.53 years, and 65.3% being women. A total of 67.8% had living with others, 35.7% completed elementary school, and 14.6% had no formal education. Among these, 61.8% reported having a religion, and 83.9% earned less than 200 (10,000 KRW) monthly. About 52.2% had 2~3 chronic diseases, and the most common perceived health status was 'unhealthy' (42.2%). Annual dementia screening plans were 'definitely planned' by 53.8%, 'uncertain' by 40.2%, and 'not planned' by 6.0% (Table 1).

Participants’ dementia prevention behaviors differed significantly according to age (F = 4.59, *p* = .008). In particular, those aged 71~80 years scored higher (60.77 ± 9.01) than did those aged 65~70 (55.91 ± 9.39). Participants with religious affiliations (59.79 ± 9.14) scored higher in dementia prevention behaviors than those without (57.16 ± 8.59). Similarly, those with definite (60.28 ± 8.35) and no dementia screening plans (64.92 ± 7.04) scored higher than those uncertain their plans (55.86 ± 9.24). No significant differences in dementia prevention behaviors were observed based on sex, living arrangements, education level, monthly income, number of chronic diseases, perceived health status, family history of dementia, experience in dementia care, and experience with dementia prevention education (Table 1).

### 2. Levels of dementia knowledge, fear, and HBM variables

Out of a maximum score of 30, participants had a mean dementia knowledge score of 18.03 ± 2.83. Out of a maximum score of 150, participants had a mean dementia fear score of 85.02 ± 23.79. Among health beliefs, cues to action (4.18 ± 0.87) and self-efficacy (3.66 ± 1.03) scored the highest, whereas perceived susceptibility scored the lowest (1.85 ± 0.92). The mean score for dementia prevention behaviors was 58.78 ± 9.00 out of a maximum score of 80 (Table 2).

### 3. Predictors of dementia prevention behaviors

Correlation analysis revealed that dementia prevention behaviors were positively associated with self-efficacy (r = .33, *p* < .001), cues to action (r = .31, *p* < .001), and perceived benefits (r = .22, *p* = .002). Conversely, they were negatively associated with perceived barriers (r = −.31, *p* < .001), perceived susceptibility (r = −.28, *p* < .001) and dementia fear (r = −.16, *p* = .028) (Table 3).

To identify factors influencing dementia prevention behaviors among community-dwelling older adults, independent variables showing significant differences on univariate analysis (age, religion, and dementia screening plans), health belief variables (perceived susceptibility, severity, benefits, barriers, cues to action, health motivation, and self-efficacy), dementia knowledge, and dementia fear were analyzed. Despite initial analyses showing no significant differences or correlations, dementia knowledge was included in the regression model based on prior research indicating its potential influence on dementia prevention behaviors [11,21,22] and to explore these factors within the HBM framework. Notably, scatter plot analysis revealed a nonlinear relationship between dementia knowledge and dementia prevention behaviors, prompting the inclusion of a quadratic term for dementia knowledge on polynomial regression analysis. Our results confirmed that the quadratic term for dementia knowledge was significant, indicating a nonlinear relationship between dementia knowledge and prevention behaviors (Table 4).

To verify the assumptions of the regression analysis, residual normality was examined using the Shapiro–Wilk test (*p* = .250), confirming that residuals followed a normal distribution. The Durbin-Watson statistic was 1.95 was obtained, indicating no autocorrelation between the error terms. Homoscedasticity was verified using the Breusch–Pagan test (*p* = .716~ .771). Multicollinearity was assessed using the variance inflation factor (VIF) and tolerance values. Initially, the VIF and tolerance values for dementia knowledge and its quadratic term suggested multicollinearity (VIF ≥ 10, tolerance < 0.1). To address multicollinearity, we implemented mean centering for these variables. Mean centering is a useful technique to reduce multicollinearity in regression models by adjusting the values of independent variables [23]. As a result, the VIF values were effectively reduced to between 1.01 and 2.59, and the tolerance values improved to between 0.39 and 0.99, successfully resolving the issues of multicollinearity.

Hierarchical polynomial regression was performed to evaluate the explanatory power of dementia knowledge and fear, alongside health belief variables, for dementia prevention behaviors, with the results being summarized in Table 4. In Model 4, perceived barriers emerged as the strongest negative predictor (β = −.33, *p* < .001), indicating that lower perceived barriers corresponded to higher levels of dementia prevention behaviors. Self-efficacy (β = .31, *p* < .001) and cues to action (β = .17, *p* = .028) were identified as positive predictors, suggesting that higher levels of self-efficacy and cues to action were associated with increased dementia prevention behaviors. Participants aged 71~80 years (β = .15, *p* = .041) exhibited higher prevention behavior levels than did those aged 65~70 years. The quadratic term for dementia knowledge (β = −.14, *p* = .021) indicated that the positive influence of dementia knowledge on prevention behaviors diminished beyond a certain threshold.

## DISCUSSION

This study was conducted to identify the expansion of the HBM by assessing the impact of dementia knowledge and fear on dementia prevention behaviors among community-dwelling older adults. Using the Korean version of the Dementia Knowledge Scale [14], this study found that community-dwelling older adults had an average dementia knowledge score of 18.03 out of 30. A similar study in China among adults aged 65 and older reported scores ranging from 17.2 to 17.5 [24]. Our participants exhibited higher dementia knowledge, possibly due to increased concern about aging and the impact of public dementia awareness and prevention programs, including the National Responsibility for Dementia System launched in 2017 [25]. Previous research has shown a significant positive correlation between dementia knowledge and preventive behaviors, with higher levels of knowledge having been associated with improved adherence to prevention practices [26,27]. Considering the positive relationship between dementia knowledge and preventive behaviors, developing and expanding ongoing educational programs that focus on dementia prevention is imperative.

Dementia fear is an important psychological factor influencing dementia prevention [28]. The current study assessed dementia fear using the K-FADS [16], with participants having an average dementia fear of 85.02 out of a maximum score of 150 points. Interestingly, the average score obtained in this study was notably higher than that 53.4 points reported in a study involving adults with an average age of 57.4 years [28]. These findings suggest that fear of dementia intensifies with age, a trend supported by research conducted by Cantegreil-Kallen and Pin [29], which revealed a similar correlation between advancing age and heightened fear of dementia. Excessive dementia fear, characterized by vague negative emotions, has been found to adversely affect preventive behaviors [30]. These results underscore the necessity of providing accurate information and psychological support to community-dwelling older adults in order to mitigate dementia fear and promote effective prevention practices.

Our participants had an average dementia prevention behavior score of 58.78 out of a maximum score of 80 (equivalent to a score of 73.49 out of 100), which was higher than the average score of 69.4 out of 100 reported in a study involving high-risk older adults [9], but lower than the score of 74.11 out of 100 reported in other studies using the tools developed by Lee et al. [21] among community-dwelling older adults [31]. Such differences in scoring may be attributed to variations in the assessment tools. Indeed, Lee et al. [21] used a 3-point Likert scale to measure the subjective frequency of dementia prevention behaviors, whereas the tool used in the current study employed a 5-point Likert scale to assess behavior frequency on a weekly basis, providing a more objective measure [9].

The current study identified significant differences in dementia prevention behaviors among community-dwelling older adults according to age, religion, and dementia screening plans. Dementia prevention behaviors were highest among those aged 71~80 years, likely due to increased awareness and engagement in preventive activities with aging. Similar trends have been observed in prior studies [32,33], including research on hypertension, where older adults demonstrated more frequent health behaviors [34]. Given aging as a major dementia risk factor [35], older adults may prioritize prevention to maintain cognitive health [36]. Religious affiliation was also associated with higher dementia prevention behaviors, potentially due to social support and access to prevention information during religious gatherings. Religious engagement enhances social interaction, stress management, and knowledge-sharing, aligning with previous findings linking religion to increased dementia prevention behaviors [32,37,38]. Additionally, individuals with definite dementia screening plans exhibited higher prevention behaviors. Notably, those without screening plans also engaged in preventive behaviors, possibly due to confidence in their lifestyle choices or lower perceived benefits of screening. Raising awareness of the importance of regular dementia screenings and providing targeted support programs can further enhance prevention efforts.

After examining the relationships between dementia knowledge, fear, health beliefs, and dementia prevention behaviors in community-dwelling older adults, this study found that dementia fear, perceived susceptibility, and perceived barriers were significantly negatively correlated with dementia prevention behaviors. While perceived susceptibility is generally associated with increased health behaviors, our findings, along with previous studies [39,40], suggest the opposite trend. This may reflect optimistic thinking, where individuals engaging in active health behaviors perceive themselves at lower risk of developing dementia. Additionally, participants who already practice effective health behaviors may feel less vulnerable to dementia, leading to a weaker perceived need for additional prevention efforts. These findings highlight the complex relationship between risk perception and preventive behaviors, suggesting that tailored interventions are needed to balance awareness of susceptibility with motivation for proactive dementia prevention. Meanwhile, we found that perceived benefits, cues to action, and self-efficacy were positively correlated with dementia prevention behaviors. This finding suggests that individuals who perceive greater benefits from dementia prevention, had higher social influence encouraging preventive actions, and had greater confidence in their ability to adopt health-promoting behaviors were more likely to engage in dementia prevention behaviors. Perceived benefits, a critical motivational factor, can facilitate dementia prevention behaviors by emphasizing the improved quality of life and autonomy associated with prevention efforts [41]. The positive correlation between dementia prevention behaviors and a high level of cues to action also underscores the need to enhance social support networks for older adults, such as community-based initiatives aimed at dementia prevention and management. Based on Bandura’s self-efficacy theory, self-efficacy emphasizes an individual's belief in their ability to successfully perform a specific behavior, thereby playing a crucial role in behavior execution [42]. Thus, programs that support specific behavioral goals for dementia prevention and offer positive feedback on achievement may be effective.

Based on the theoretical framework of the HBM, the current study evaluated the impact of adding dementia knowledge and fear to the independent variables of the HBM on its explanatory power of dementia prevention behaviors. Hierarchical polynomial regression analysis was conducted to account for the nonlinear effects of dementia knowledge. Notably, our results showed that adding health belief variables to Model 2, which initially only included the demographic characteristics of the participants in Model 1, promoted a significant 23.0% increase in the adjusted R^2^ value. This finding suggests that the HBM provides a robust theoretical foundation for understanding behavioral changes related to dementia prevention, partially supporting the results of previous studies showing that perceived barriers, benefits, and severity influence dementia prevention behaviors [18,30,41]. In particular, perceived barriers and self-efficacy were identified as significant predictors of dementia prevention behaviors. One study found that perceived barriers negatively influenced behaviors, indicating that higher perceived difficulties in performing specific actions increased the likelihood of avoidance [43]. This finding highlights the need for strategies that identify and reduce the challenges older adults face in initiating and maintaining dementia prevention behaviors. Werner [44] emphasized that misconceptions about dementia and a lack of knowledge about prevention could act as barriers to behavioral change. Thus, providing accurate information about dementia prevention and management tailored to the functional status of older adults in accessible settings, such as senior centers or welfare facilities, is crucial. The current study reaffirmed self-efficacy as a key factor influencing dementia prevention behaviors, consistent with previous research [21]. Higher self-efficacy has been associated with increased engagement in dementia prevention behaviors [22], suggesting that enhancing self-efficacy in older adults, particularly community-dwelling seniors, could positively impact dementia prevention efforts. Evidence shows that self-efficacy is influenced by individual differences in performance capabilities and is crucial for adapting to and coping with diverse situations [45]. Previous studies have consistently identified self-efficacy as a primary factor in maintaining long-term dementia prevention behaviors [18,22,33]. Therefore, incorporating the strategies proposed by Bandura [46], such as mastery experiences, vicarious experiences, and verbal persuasion, into programs aiming to enhance self-efficacy in older adults is essential.

Model 4, which incorporated dementia health beliefs, knowledge, fear, and the quadratic term of dementia knowledge, significantly increased explanatory power by 35.5%. This study contributes to expanding the HBM by demonstrating that dementia knowledge and fear are critical variables in understanding dementia prevention behaviors. Unlike traditional HBM models that focus solely on perceived susceptibility, severity, benefits, and barriers, our findings highlight how cognitive and emotional factors—specifically dementia knowledge and fear—shape preventive behaviors. The inclusion of a quadratic term for dementia knowledge further refines the model by revealing its nonlinear relationship with dementia prevention behaviors. While increased dementia knowledge initially promotes preventive behaviors, excessive knowledge may heighten dementia fear, leading to avoidance behaviors or reduced motivation for prevention efforts. This underscores the need for tailored educational interventions that balance knowledge dissemination with psychological well-being to optimize dementia prevention strategies.

These findings align with previous research suggesting that excessive or inaccurate dementia knowledge can heighten dementia fear, delay diagnosis, and hinder treatment, ultimately negatively affecting prevention and management efforts [47,48]. Smith et al. [49] highlighted that mild dementia fear can motivate preventive behaviors, whereas extreme fear may obstruct them. Similarly, Bowen et al. [50] noted that dementia fear can either negatively impact dementia-related health behaviors or enhance sensitivity to dementia prevention through increased awareness. This study contributes to the growing body of research emphasizing that dementia prevention efforts must consider not only cognitive factors but also psychological and emotional influences. Future research should explore how dementia knowledge and fear interact to influence behavior, particularly within the HBM framework.

From a biological nursing science perspective, this study provides critical insights into dementia prevention behaviors among older adults. By integrating psychological and cognitive components into health behavior models, the findings support the development of holistic nursing interventions. Despite its contributions, this study has limitations. First, it was conducted in a single region, limiting the generalizability of the findings to other populations. Second, the cross-sectional design prevents causal inferences, and the self-reported nature of dementia prevention behaviors may introduce response biases. Third, while participants were screened for cognitive impairment through the CIST conducted by the Jeju Center for Dementia within the year prior to recruitment, cognitive status may change over time. This potential variation in cognitive function was not reassessed at the time of data collection, which may influence participants’ dementia-related perceptions and behaviors. Future studies should employ longitudinal designs, reassess cognitive function at multiple time points, and include diverse populations to further validate the expanded HBM framework incorporating dementia knowledge and fear.

## CONCLUSION

Hierarchical polynomial regression analysis demonstrated significant results across all models: Model 1, including demographic variables; Model 2, with added health belief components; Model 3, with centered dementia knowledge and dementia fear; and Model 4, including a quadratic term for dementia knowledge. The explanatory power of the HBM ranged from 11.8% to 35.5. Significant improvements in adjusted R^2^ values were observed for Models 2 and 4 but not for Model 3.

In Model 4, perceived barriers emerged as the most significant negative predictor of dementia prevention behaviors, indicating that lower perceived barriers increased engagement in dementia prevention behaviors. Meanwhile, self-efficacy and cues to action were identified as positive predictors of dementia prevention behaviors. The quadratic term for dementia knowledge suggested that dementia knowledge had a diminishing positive effect on prevention behaviors beyond a certain threshold. To develop more comprehensive models and better understand their combined effects on dementia prevention behaviors, future studies should investigate the interaction effects of dementia knowledge and dementia fear on prevention behaviors.

## Figures and Tables

**Table 1.** Differences in Dementia Prevention Behaviors According to Participants’ Characteristics (N = 199)

Variables	Categories	n (%)	M ± SD	t/F	*p*
Age (yr)	65~70	58 (29.1)	55.91 ± 9.39^a^	4.59	.008 (a < b^†^)
	71~80	73 (36.7)	60.77 ± 9.01^b^		
	≥ 81	68 (34.2)	59.10 ± 8.10^c^		
Sex	Men	69 (34.7)	58.42 ± 9.88	−0.41	.679
	Women	130 (65.3)	58.98 ± 8.53		
Living arrangements	Living alone	64 (32.2)	58.13 ± 9.42	1.50	.135
	Living with others	135 (67.8)	60.17 ± 7.95		
Religion	Yes	123 (61.8)	59.79 ± 9.14	2.02	.045
	No	76 (38.2)	57.16 ± 8.59		
Education level	< Elementary school	29 (14.6)	97.07 ± 7.73	1.61	.189
	Elementary school	71 (35.7)	59.49 ± 8.96		
	Middle school	43 (21.6)	56.91 ± 9.13		
	≥ High school	56 (28.1)	60.21 ± 9.40		
Average monthly income (10,000 KRW)	< 200	167 (83.9)	58.95 ± 8.61	0.51	.611
	≥ 200	32 (16.1)	57.91 ± 10.90		
Number of chronic diseases	0~1	65 (32.7)	58.34 ± 8.50	1.39	.253
	2~3	104 (52.2)	59.66 ± 9.20		
	≥ 4	30 (15.1)	56.70 ± 9.23		
Perceived health status	Unhealthy	84 (42.2)	58.32 ± 9.47	0.20	.817
	Average	67 (33.7)	58.93 ± 9.72		
	Healthy	48 (24.1)	59.35 ± 7.05		
Family history of dementia	No	159 (79.9)	59.09 ± 9.32	−0.97	.333
	Yes	40 (20.1)	57.55 ± 7.59		
Experience in dementia care	No	160 (80.4)	58.74 ± 9.10	0.15	.883
	Yes	39 (19.6)	58.97 ± 8.69		
Experience in dementia prevention education	No	109 (54.8)	58.24 ± 8.77	−0.94	.348
	Yes	90 (45.2)	59.44 ± 9.28		
Dementia screening plan	Definitely planned	107 (53.8)	60.28 ± 8.35^a^	9.18	< .001 (a,c > b^†^)
	Uncertain	80 (40.2)	55.86 ± 9.24^b^		
	Not planned	12 (6.0)	64.92 ± 7.04^c^		

M = Mean; SD = Standard deviation; KRW = Korean won.

† Scheffé's test.

**Table 2.** Levels of Dementia Knowledge, Dementia Fear, Dementia Health Beliefs and Dementia Prevention Behaviors of Participants (N = 199)

Variables (range)		M ± SD	Min	Max
Dementia knowledge (0~30)		18.03 ± 2.83	11.00	28.00
Dementia fear (30~150)		85.02 ± 23.79	43.00	130.00
Dementia health beliefs (1~5)	Perceived susceptibility	1.85 ± 0.92	1.00	5.00
	Perceived severity	2.50 ± 0.88	1.00	5.00
	Perceived benefits	3.62 ± 1.04	1.00	5.00
	Perceived barriers	2.32 ± 1.05	1.00	5.00
	General health motivation	3.04 ± 0.91	1.00	5.00
	Cue to action	4.18 ± 0.87	1.25	5.00
	Self-efficacy	3.66 ± 1.03	1.00	5.00
Dementia prevention behaviors (16~80)		58.78 ± 9.00	35.00	76.00

M = Mean; SD = Standard deviation; Min = Minimum; Max = Maximum.

**Table 3.** Correlations between Dementia Knowledge, Dementia Fear, Dementia Health Beliefs and Dementia Prevention Behaviors (N = 199)

Variables	1	2	3	4	5	6	7	8	9	10
	r (*p*)									
1. Dementia knowledge	1	-	-	-	-	-	-	-	-	-
2. Dementia fear	.07 (.313)	1	-	-	-	-	-	-	-	-
3. Perceived susceptibility	−.16 (.021)	.43 (< .001)	1	-	-	-	-	-	-	-
4. Perceived severity	−.06 (.435)	.72 (< .001)	.47 (< .001)	1	-	-	-	-	-	-
5. Perceived benefits	.22 (.002)	.21 (.004)	−.10 (.182)	.19 (.006)	1	-	-	-	-	-
6. Perceived barrier	−.15 (.033)	.37 (< .001)	.20 (.005)	.34 (< .001)	.05 (.478)	1	-	-	-	-
7. Cue to action	.16 (.028)	.22 (< .001)	−.17 (.018)	.07 (.364)	.52 (< .001)	.11 (.127)	1	-	-	-
8. General health motivation	.08 (.245)	.26 (< .001)	−.09 (.209)	.21 (.003)	.39 (< .001)	.33 (< .001)	.39 (< .001)	1	-	-
9. Self-efficacy	.05 (.493)	.14 (.050)	−.17 (.016)	.07 (.353)	.45 (< .001)	.23 (.001)	.57 (< .001)	.41 (< .001)	1	-
10. Dementia prevention behaviors	−.01 (.905)	−.16 (.028)	−.28 (< .001)	−.12 (.082)	.22 (.002)	−.31 (< .001)	.31 (< .001)	−.01 (.935)	.33 (< .001)	1

**Table 4.** Factors Affecting Participants’ Dementia Prevention Behaviors (N = 199)

Variables	model 1				model 2				model 3				model 4			
	B	β	t	*p*	B	β	t	*p*	B	β	t	*p*	B	β	t	*p*
(Constant)	52.22		35.50	< .001	47.87		13.69	< .001	48.37		13.85	< .001	49.06		14.17	< .001
Age (yr)^†^ (ref. = 65~70)																
71~80	4.04	.22	2.66	.009	2.95	.16	2.13	.034	2.85	.15	2.07	.039	2.81	.15	2.06	.041
≥ 81	2.24	.12	1.44	.152	1.11	.06	0.79	.429	1.45	.08	1.03	.302	1.46	.08	1.05	.295
Religion^†^ (ref. = no)																
yes	2.87	.16	2.31	.022	2.03	.11	1.82	.070	1.93	.11	1.75	.082	1.75	.10	1.60	.112
Dementia screening plan^†^ (ref. = uncertain)																
Definitely planned	3.71	.21	2.88	.004	2.37	.13	1.99	.049	2.64	.15	2.22	.028	2.32	.13	1.96	.051
Not planned	9.13	.24	3.46	.001	5.98	.16	2.47	.014	4.70	.12	1.89	.060	4.77	.13	1.95	.053
Perceived susceptibility					−1.03	−.11	−1.45	.148	−0.99	−.10	−1.37	.173	−1.02	−.10	−1.42	.157
Perceived severity					0.26	.03	0.33	.739	0.75	.07	0.80	.422	0.72	.07	0.78	.437
Perceived benefits					0.16	.02	0.24	.809	0.38	.04	0.58	.563	0.29	.03	0.44	.660
Perceived barriers					−2.59	−.30	−4.39	< .001	−2.68	−.31	−4.47	< .001	−2.85	−.33	−4.77	< .001
Cue to action					1.52	.15	1.90	.060	1.79	.17	2.20	.029	1.79	.17	2.22	.028
General health motivation					−1.10	−.11	−1.57	.119	−1.02	−.10	−1.47	.143	−0.98	−.10	−1.42	.157
Self-efficacy					2.81	.32	4.17	< .001	2.63	.30	3.92	< .001	2.75	.31	4.12	< .001
Dementia knowledge^‡^									−0.35	−.11	−1.72	.088	−0.32	−.10	−1.59	.114
Dementia fear									−0.04	−.10	−1.08	.283	−0.03	−.08	−0.81	.417
Dementia knowledge^2§^													−0.11	−.14	−2.33	.021
R^2^	.14				.37				.39				.40			
Adj. R^2 ^(△ Adj. R^2^)	.12				.33 (.23^∥^)				.34 (.02)				.36 (.02^∥^)			
F(*p*)	6.31(< .001)				9.13(< .001)				8.28(< .001)				8.27(< .001)			
Durbin-Watson = 1.95, Tolerance = 0.39~0.99, VIF = 1.01~2.59																

ref. = Reference; Adj. = Adjusted; VIF = Variance inflation factor.

† dummy variable;

‡ centered dementia knowledge;

§ centered quadratic term for dementia knowledge;

∥ significant change in R^2^ (*p* < .001, hierarchical polynomial regression).

## Data Availability

The data that support the findings of this study are available from the corresponding author upon reasonable request.
